# Antiviral effects of Atractyloside A on the influenza B virus (Victoria strain) infection

**DOI:** 10.3389/fmicb.2022.1067725

**Published:** 2023-01-10

**Authors:** Jicheng Han, Xiangyu Zhu, Zihan Gao, Yan Xiao, Jinxin Zhang, Peng Wang, Jinbo Fang, Yiquan Li, Yilong Zhu, Yue Li, Ningyi Jin, Huijun Lu, Dazhuan Lin, Wenshen Liu

**Affiliations:** ^1^Changchun Veterinary Research Institute, Chinese Academy of Agricultural Sciences, Changchun, China; ^2^Academician Workstation, Changchun University of Chinese Medicine, Changchun, China; ^3^College of Animal Science and Technology, Jilin Agricultural University, Changchun, China; ^4^College of Pharmaceuticals and Food, Changchun Medical College, Changchun, China

**Keywords:** Atractyloside A, antiviral drugs, macrophage polarization, influenza virus, influenza B virus (IBV)

## Abstract

Influenza viruses pose a serious threat to human health, infecting hundreds of millions of people worldwide each year, resulting in a significant increase in global morbidity and mortality. Influenza activity has declined at the onset of the COVID-19 pandemic, but the genetic diversity of B/Victoria lineage viruses has increased significantly during this period. Therefore, the prevention and treatment of the influenza B Victoria strain virus should continue to attract research attention. In this study, we found that Atractyloside A (AA), one of the effective components in Atractylodes lancea (Thunb.) DC shows potential antiviral properties. This study shows that AA not only possesses anti-influenza B virus infection effects *in vivo* and *in vitro* but also can regulate macrophage polarization to the M2 type, which can effectively attenuate the damage caused by influenza B virus infection. Therefore, Atractyloside A may be an effective natural drug against B/Victoria influenza infection.

## Introduction

Influenza is an acute respiratory infection caused by the influenza virus (Huang et al., 2022). There are four types of influenza viruses: A, B, C, and D (Yamayoshi and Kawaoka, 2019); of these, A and B tend to cause seasonal epidemics (Petrova and Russell, 2018). Influenza B viruses are currently divided into two antigenically distinct lineages, Victoria and Yamagata. Unlike influenza A virus subtypes that have periodically emerged from animals caused pandemics and circulated as single major antigenic variants, the Victoria and Yamagata lineages emerged as antigenic variants after the differentiation of influenza B viruses in the early 1980s. Since 2001, influenza B began to circulate globally (Shaw et al., 2002). Currently, the emergence of a large number of mutant influenza B viruses has been reported worldwide, indicating significant changes in the evolution and epidemiology of the influenza B viruses (Huang et al., 2022). Therefore, influenza B should be prominently monitored on the global health radar. Influenza activity declined sharply at the onset of the COVID-19 pandemic and gradually increased in 2021, but the intensity of activity remained lower than before the COVID-19 pandemic (Huang et al., 2022). Since the emergence of COVID-19, the diversity of circulating influenza virus (sub)types has decreased compared to previous seasons and almost exclusively involves the B/Victoria lineage viruses, while the genetic diversity of B/Victoria lineage viruses has increased significantly during this period (Huang et al., 2022).

Safe and effective vaccines and antiviral drugs are available for influenza prevention and treatment. Increasing influenza vaccination rates can significantly reduce influenza-related morbidity and mortality (Paules and Subbarao, 2017; Feng et al., 2018). In 2012, the World Health Organization (WHO) began recommending a quadrivalent influenza vaccine, which contains two lineages of influenza B viruses; however, influenza vaccines have so far failed to fundamentally successfully block or eliminate the spread of influenza viruses in the human population. On the one hand, this stems from the suboptimal immunogenicity of the vaccine itself (Jackson et al., 2017; Uyeki, 2017; Paules et al., 2018). On the other hand, other factors are involved, such as the mismatch between the vaccine and circulating strains (Belongia et al., 2016), and vaccination timing (Ferdinands et al., 2017). Therefore, the development of effective antiviral drugs against the influenza virus is necessary. Currently, there are three classes of antiviral drugs used to treat influenza: transmembrane ion channel (M2 protein) blockers, neuraminidase inhibitors, and a cap-dependent endonuclease inhibitor that interferes with viral RNA transcription and blocks viral replication (Han et al., 2018). The WHO recommends the prompt administration of oseltamivir as a first-line treatment for those with suspected or confirmed influenza virus infection or those at risk of severe illness. However, during the 2017–2018 influenza epidemic, the proportion of patients aged 14 years or older treated with oseltamivir was significantly reduced and the clinical manifestations of the disease were more severe. Therefore, it is necessary to develop high-yield and effective anti-seasonal influenza drugs.

Drug repurposing is a common strategy used in antiviral drug research to achieve rapid applications during times of public emergency (Al-Karmalawy et al., 2021). AA is an important natural compound present in Atractylodes lancea (Thunb.) DC (AL). Studies have shown that AA exhibits various pharmacological effects including antihypertensive, antiglycemic, anti-tumor, and intestinal mucosal barrier protection (Tu et al., 2020). The effects of AA on the influenza virus have not yet been reported. In this study, AA was tested for its anti-influenza properties against infection with the B Victoria strain and to examine its *in vivo* effects on influenza B virus-related pneumonia. We found that AA exhibits anti-influenza virus activity *in vitro* and *in vivo* and activates macrophages *in vivo*, thereby effectively improving the potential damage of influenza virus pneumonia on the body. As such, AA is a potential anti-influenza B virus drug.

## Materials and methods

### Cells and animals

The human lung cancer cell line A549 and the canine kidney cell line MDCK were purchased from BeNa Culture Collection. A549 and MDCK cells were cultured in RPMI 1640 and DMEM medium containing 10% FBS, respectively. Influenza B virus (IBV) was stored in the Changchun Veterinary Research Institute, Chinese Academy of Agricultural Sciences. Atractyloside A (AA) was purchased from MedChemExpress (Cat#HY-N0237). The C57BL/6 mice used in this study were purchased from Beijing Wei Tong Li Hua Laboratory Animal Technology Co., Ltd.

### Cell viability assay

A549 or MDCK cells were cultured in a 96-well plate (1 × 10^4^ cells/well) in an incubator at 37°C and 5% CO_2_ for 12 h and then treated with different concentrations of Atractyloside A (AA); four replicate wells were used for each test group. After treatment for 24 h and 48 h, CCK-8 was added and cells were further cultured for 2 h; the OD450 value was detected, and cell viability was calculated.

### Half maximal inhibitory concentration assay

MDCK cells were plated in 96-well plates and assigned to an IBV group, IBV+ different concentrations of AA (50, 75, 100, 125, and 150 μM) group, or a control group. Seventy-two hours after infection of the MDCK cells with 0.05 MOI IBV, half maximal inhibitory concentration assay (IC50) was obtained by colorimetric CCK-8 assay and calculated using non-linear regression analysis in GraphPad Prism 9 software.

### Immunofluorescence analysis

A549 or MDCK cells were plated in 12-well plates and assigned to an IBV group, an IBV+AA group, or a control group. Each experimental group was treated with the corresponding solution and placed in a cell incubator for 24 h. Immunofluorescence assays were then performed. After incubation, the culture mixture was discarded and cells were fixed with 4% paraformaldehyde for 30 min, treated with 0.1% Triton X-100, and blocked with 5% bovine serum albumin (BSA). After blocking, the cells were stained with primary antibody for 2 h at room temperature and then stained with fluorescent secondary antibody for 1 h in the dark. Finally, the samples were stained with DAPI solution for 10 min before observation. The antibody used in this study was the Anti-Influenza B Virus Nucleoprotein from Abcam (Cat#ab20711).

### Virus yield reduction assay

After the A549 or MDCK cells were plated in 12-well plates and incubated for 12 h, 0.1 MOI of IBV was added with different concentrations of AA. Twenty-four hours after IBV infection + treatment, cellular RNA was extracted and subjected to the quantitative real-time PCR (qPCR) utilizing a quantitative PCR instrument (ABI7500). The plasmid pUC57-HA (2896bp) was constructed based on the HA gene of IBV, and the plasmid was diluted to 10^2^-10^9^ copies as a standard to calculate the viral copy number after IBV infection. The primers used in this study were 5′-ATTTTGCAAAYCTCAAAGGAACA-3′ (Forward) and 5′-TTGTTCTRTCGTGCATTATAGG-3′ (Reverse). The IBV-Victoria probe used in this study was 5′-VIC-TGGGYAGACCAAAATGCACRG-BHQ1-3′.

### Hemagglutination assay (HA)

After the A549 or MDCK cells were plated in 12-well plates and incubated for 12 h, 0.1 MOI of IBV was added with different concentrations of AA. Twenty-four hours after IBV infection + treatment, the culture supernatant was collected for a hemagglutination assay (HA). In 12 wells of a 96-well V-shaped microplate, 25 μl of PBS was added to each well. Twenty-five microliters of cell culture solution were added to the first well and then serially diluted 10 times. After serial multiple dilutions, 25 μl of 1% chicken red cell suspension was added to each well, mixed well, and incubated for 40 min at room temperature for result determination.

### Western blot

A549 cells were inoculated with 0.1 MOI of IBV, and the IBV+AA treatment group and control group were started simultaneously. Twenty-four hours after infection of the A549 cells with IBV, the cells were collected, and total proteins were extracted from the cells. Each group sample was loaded at 25 μg of total protein for detection. After SDS-PAGE was transferred to a PVDV membrane and blocked at room temperature for 2 h, the corresponding primary antibody was added for incubation at 4°C overnight. Then, the membrane was incubated with goat anti-rabbit or goat anti-mouse secondary antibody for 50 min. The primary antibodies used in this study were the Anti-Influenza B Virus Nucleoprotein from Abcam (Cat#ab20711) and the Rig-I Pathway Antibody Sampler kit from Cell Signaling Technology (Cat#8348).

### Luciferase assay

A total of 293 cells were transfected together with pIFN-β-Luc plasmids and Renilla plasmids (pRL-TK). Two hundred ninety-three cells were infected with 0.05 MOI of IBV before harvesting, together with the addition of AA (40 μM) as the intervention. The luciferase activity was tested using the Dual-Lumi™ Luciferase Assay kit (Beyotime, RG088M) after IBV infection for 24 h. pIFN-β-Luc (Cat#ZT206) and pRL-TK (Cat#BR018) plasmids were from the Hunan Fenghui Biotechnology Co., Ltd.

### *In vivo* challenge

Six-week-old female C57BL/6 SPF-grade mice were used for intranasal inoculation with 50 μl of PBS containing 1 × 10^4.5^ TCID_50_ of IBV. Animals were divided into four groups (six mice for each group): an IBV infection group, an IBV infection + AA treatment group (10 mg/kg and 30 mg/kg), and a control group. AA was administered orally over 14 consecutive days, starting 1 day before IBV inoculation. IBV infection and control groups were orally treated with a placebo solution (0.2 ml saline) over 14 consecutive days. Body weight, food intake, and mortality were then monitored; death was defined at ≥35% weight loss. Fourteen days after the IBV infection, the animals were euthanized, and the lung tissue was collected and placed in tissue fixative for histopathological analysis. The fixed lung tissue was utilized for HE staining, immunohistochemical analysis using the nucleoprotein of the IBV antibody, and immunofluorescence analysis using the F4/80 and the nucleoprotein of the IBV antibodies. Analysis software (Image-Pro Plus 6.0, Media Cybernetics) was used to analyze the average signal density of stained samples. The analysis software analyzes each image to derive the IOD (integrated optical density) value for each area (pixel area of tissue). The average optical density value IOD/area (Mean Density) was also derived. The antibodies used in this study were the Anti-Influenza B Virus Nucleoprotein antibody from Abcam (Cat#ab20711) and the Anti-F4/80 antibody from Cell Signaling Technology (Cat# 70076S).

### Macrophage activation and polarization

To test the ability of AA to activate macrophages, we used 6-week-old SPF-grade C57BL/6 mice to isolate and obtain primary bone marrow-derived cells. For the specific experimental methods, we refer to previous studies (Han et al., 2021). The obtained primary macrophages were plated and incubated in a 6-well plate for 8 h and then separated into three groups: Atractyloside A, LPS, and the mock control. After adding the corresponding treatment solution, each experimental group was placed in a 37°C cell incubator for 48 h. After incubation, the cells were collected and stained with APC-F4/80 (BioLegend, Cat#157306), FITC-MHCII (BioLegend, Cat#107696), FITC-CD40, PE-CD80 (BioLegend, Cat#104708), and FITC-CD86 (BioLegend, Cat#105006) flow antibodies for flow cytometry analysis to identify macrophage phenotypes.

To further analyze the effect of AA on macrophage polarization *in vivo*, IBV-infected mice were euthanized after 7 days and mononuclear cells in the lungs were isolated and analyzed by flow cytometry. Mononuclear cells were obtained from lung tissue isolated from three mice for flow-through antibody staining. Then, the isolated cells were stained with APC-F4/80 (BioLegend, Cat#157306), Percp-CD11c (BioLegend, Cat#117326), and FITC-CD206 (BioLegend, Cat#141704) flow antibodies for the flow cytometry analysis.

### Statistical analysis

All experiments were performed at least three times independently. Data are presented as mean ± standard deviation (SD) and compared between groups using *t*-tests. Univariate and multivariate ANOVAs were used to calculate between-group differences. A *p* < 0.05 was used to determine significance; significance levels are presented as ^*^p < 0.05, ^**^p < 0.01, ^***^p < 0.001, and ^****^p < 0.0001.

## Results

### Atractyloside A shows antiviral effects against IBV infection *in vitro*

**Figure 1:**
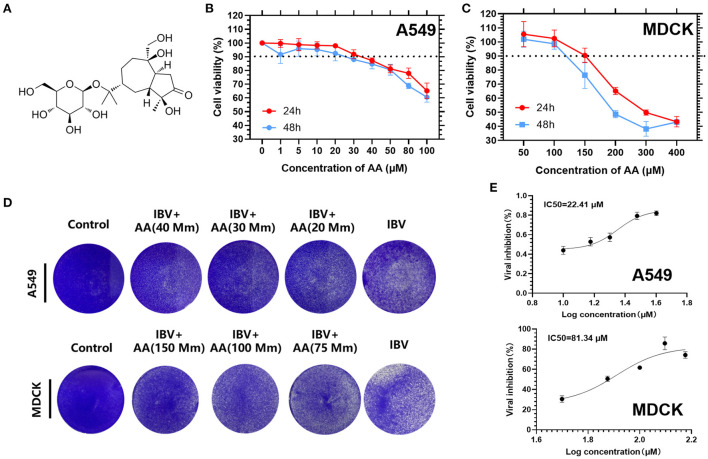
Cell viability of Atractyloside A on MDCK and A549 cells. **(A)** Chemical structure of Atractyloside A. Cell viability of Atractyloside A on A549 **(B)** and MDCK **(C)** cells. **(D)** Crystal violet staining of MDCK and A549 cells infected with IBV, or with Atractyloside A +IBV, or mock control. **(E)** Half maximal inhibitory concentration assay (IC_50_).

MDCK and A549 cells were plated on 96-well plates with various concentrations of AA (Figure 1A) and incubated in a cell culture incubator at 37°C. Cell viability assays were conducted by a colorimetric CCK-8 assay after 24 h and 48 h. We found that A549 cells treated with 30 uM AA had over 90% cell viability at 24 h (Figure 1B) and MDCK cells treated with 100 uM AA had over 90% cell viability at 24 h and 48 h (Figure 1C). Cytotoxic concentration 50 (CC_50_) and cytotoxic concentration 90 (CC_90_) for each group were calculated using GraphPad Prism 9 software. At 24 h, the CC_90_ of A549 and MDCK cells were 829.9 μM and 265.9 μM, respectively. The CC_50_ of A549 and MDCK cells were 184.1 μM and 180.7 μM, respectively. MDCK and A549 cells were then plated on 12-well plates, infected with 0.1 MOI of IBV, and incubated with AA for 48 h. After incubation, crystal violet staining was performed (Figure 1D). MDCK cells were then plated on 96-well plates, infected with 0.05 MOI of IBV, and incubated with AA for 72 h. After incubation, half maximal inhibitory concentration assay was obtained by colorimetric CCK-8 assay, showing that the IC_50_ for A549 and MDCK cells were 22.4 μM and 81.34 μM (Figure 1E).

**Figure 2:**
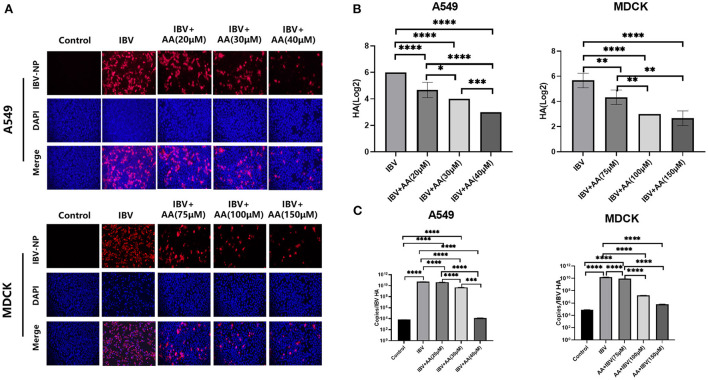
Antiviral effects of Atractyloside A against IBV infection *in vitro*. MDCK and A549 cells were infected with 0.1 MOI of IBV and treated with various concentrations of AA for 24 h. **(A)** Immunofluorescence analysis of the expression of IBV nucleoprotein after infection. Hemagglutination test **(B)** and virus copy number detection **(C)** after IBV infection. The results are presented as mean ± standard deviation (*p < 0.05, **p < 0.01, ***p < 0.001, and ****p < 0.0001).

To further explore the ability of AA to inhibit viral infection *in vitro*, we performed immunofluorescent staining, HA assay, and viral copy number analysis after IBV infection for 24 h *in vitro*. MDCK and A549 cells were infected with 0.1 MOI of IBV and treated with various concentrations of AA. Immunofluorescence was observed by a fluorescence microscope (Olympus BX53). We found that after IBV infection, with increasing AA concentration, nucleoprotein immunofluorescence decreased significantly (Figure 2A). Immunofluorescence analysis of A549 cells infected with IBV+AA for 24 h after treatment showed positive rates of 43.11%, 9.89%, 6.99%, and 4.68% for the IBV, IBV+AA (20 μM), IBV+AA (30 μM), and IBV+AA (40 μM) groups, respectively. Immunofluorescence analysis of MDCK cells showed positive rates of 98.03%, 10.54%, 7.28%, and 6.70% for the IBV, IBV+AA (75 μM), IBV+AA (100 μM), and IBV+AA (150 μM) groups, respectively.

The results of HA detection and virus copy number detection are shown in Figures 2B, C. With increasing AA concentration, HA and virus copy numbers decreased significantly. Compared with the IBV group, virus copy numbers in A549 cells decreased by 1.23 × (20 μM), 10.99 × (30 μM), and 4.17 × 10^6^× (40 μM) with increasing AA concentrations. With increased AA concentrations, virus copy numbers in MDCK cells decreased by 1.88×, 885.9×, and 2.59× 10^3^× compared with the IBV group. Compared with the IBV group, HA in A549 cells decreased by 1.28×, 1.5×, and 2× with increased AA concentrations. With increasing AA concentrations, HA in MDCK cells decreased by 1.31×, 1.89×, and 2.12× compared with the IBV group. Combined with the above results, AA could attenuate IBV infection *in vitro*.

### Atractyloside A can increase levels of phosphorylation of IRF3 to inhibit IBV replication

**Figure 3:**
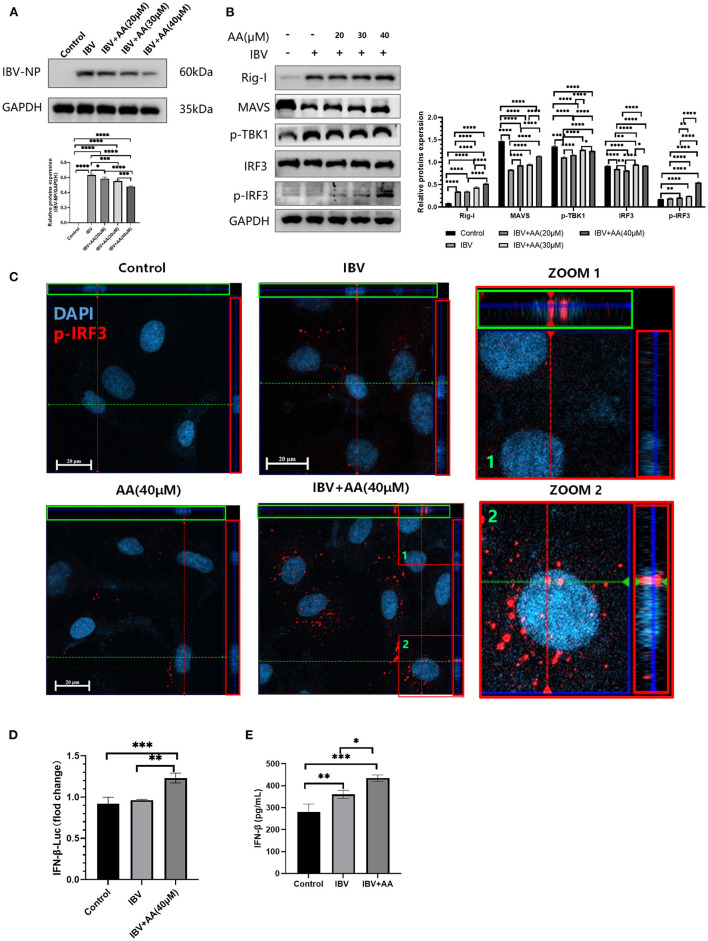
Atractyloside A can increase levels of phosphorylation of IRF3 to inhibit IBV replication. **(A)** Western blot analysis of nucleoprotein expression after IBV infection in Atractyloside A treatment groups with different drug concentrations. **(B)** Western blot analysis of RIG-I pathway proteins after IBV infection in Atractyloside A treatment groups with different drug concentrations. **(C)** Laser confocal observation of *p*-IRF3 expression after IBV infection in the different groups. **(D)** IFNβ luciferase activity was analyzed after IBV infection in the different groups. **(E)** Levels of IFN-β in the serum. The results are presented as mean ± standard deviation (*p < 0.05, **p < 0.01, ***p < 0.001, and ****p < 0.0001).

A549 cells were infected with IBV at 0.1 MOI, and various AA concentrations were added. After incubation for 24 h, A549 cells from each group were collected for protein extraction, and the extracted proteins were used to analyze changes in type I interferon pathway proteins. We found that increasing concentrations of AA reduced the expression of the IBV nucleoprotein (Figure 3A). This indicated that AA could attenuate IBV infection. To analyze the underlying mechanism, we quantified type I interferon pathway-related proteins. We found that A549 infected IBV, and with increasing AA concentrations, the levels of *p*-IRF3 were increased (Figure 3B). At the same time, we also found that AA could also increase the levels of *p*-IRF3 in the absence of IBV infection (Supplementary Figure S1). These results showed that AA can activate the production of type I interferon. For further analysis, we performed immunofluorescence analysis with images captured by confocal microscopy (Zeiss Axio observer 7). We found that AA could increase the phosphorylation of IRF3 and its nuclear translocation to activate type I interferon after IBV infection (Figure 3C). IFN-β-Luc showed that the expression of IFN-β-Luc was enhanced after adding AA (Figure 3D). We then further explored the ability of AA to activate interferon *in vivo*. We found that the IBV+AA-treated mouse group showed higher expression of IFN-β in the serum compared to the IBV-infected group (*p* < 0.05) and the control group (*p* < 0.001) (Figure 3E). This finding further validated that AA can increase IRF3 phosphorylation to inhibit IBV replication.

### Atractyloside A exhibits favorable antiviral effects against IBV infection *in vivo*

**Figure 4:**
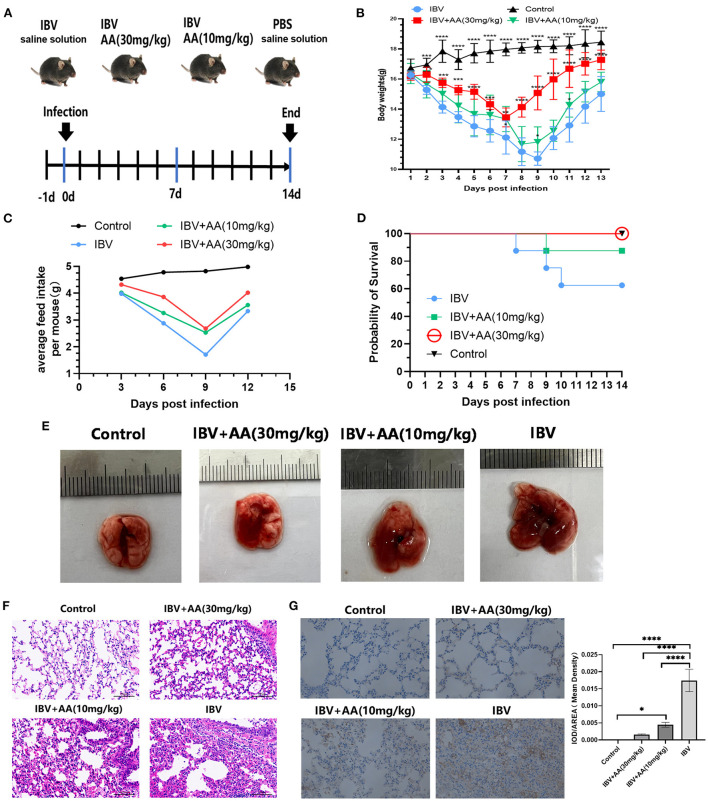
Antiviral effects of Atractyloside A against IBV infection *in vivo*. **(A)** Efficacy evaluation of Atractyloside A and infection procedure in mice. Changes in body weight **(B)** and feed intake **(C)** after infection in mice. **(D)** The survival rate of mice after infection. **(E)** Lung tissue of mice infected with IBV in the different groups. H&E **(F)** and immunohistochemical **(G)** analysis of lung tissue after infection. The results are presented as mean ± standard deviation (*p < 0.05, **p < 0.01, ***p < 0.001, and ****p < 0.0001).

To explore the anti-IBV effects of AA *in vivo*, challenge experiments were performed as shown in Figure 4A. Food intake and body weight in the IBV and AA treatment groups trended down and then up again, but the recovery in body weight was significantly faster in the 30 mg/kg AA treatment group compared to the IBV group (Figures 4B, C). Survival was 100% in the high-dose AA group, compared with 87.5% in the low-dose group and 62.5% in the IBV group (Figure 4D). The above results show that AA can effectively improve survival following influenza virus infection. To further analyze the anti-influenza virus effects of AA *in vivo*, conducted pathological analyses of the lung tissue of experimental animals. H&E staining results showed that the AA treatment group showed less tissue damage (Figure 4F). Immunohistochemical analyses of IBV nucleoprotein showed that AA could effectively reduce the replication of IBV in the lungs (Figure 4G). The aforementioned results suggest that AA can resist IBV infection *in vivo*.

### Atractyloside A can activate macrophages

**Figure 5:**
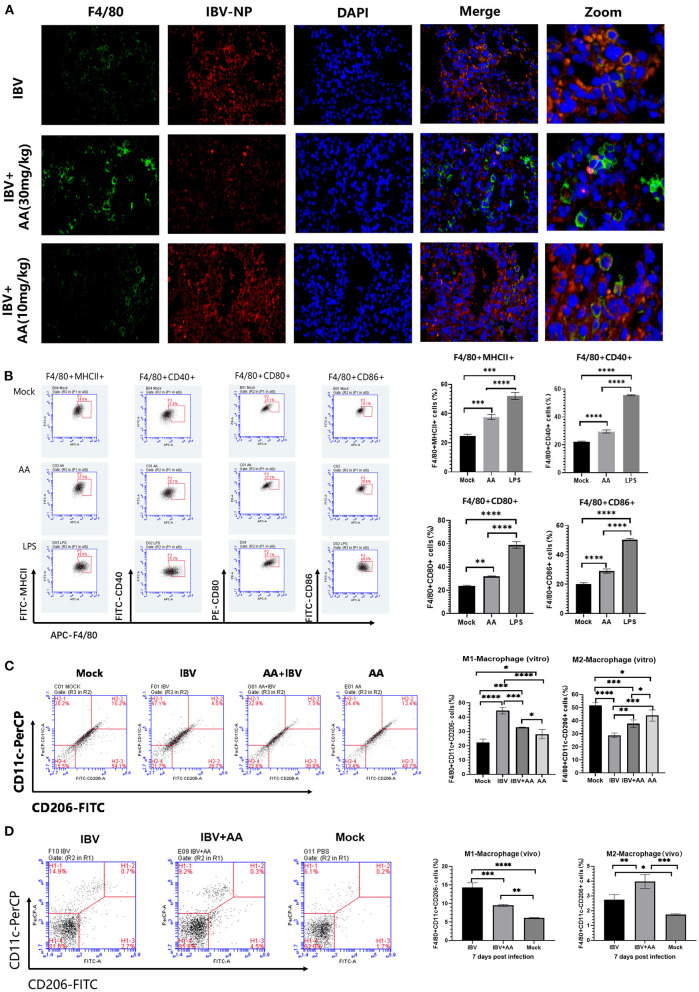
Levels of activation and polarization of macrophages. **(A)** Immunofluorescence analysis of lung tissue after infection. The intensity of green (FITC) fluorescence represents the expression of F4/80 protein, while the intensity of red (APC) fluorescence represents the expression of IBV nucleoprotein. **(B)** The analysis of Atractyloside A induced macrophage activation. Flow cytometry was performed to analyze the proportions of F4/80+MHC-II+, F4/80+CD80+, F4/80+CD40+, and F4/80+CD86+ expressing macrophages. **(C)** Effect of Atractyloside A treatment on the polarization levels of macrophages *in vitro*. Flow cytometry was performed to analyze the proportions of bone marrow-derived macrophages (BMMs). **(D)** Effect of Atractyloside A treatment on the polarization levels of macrophages in lung tissue after IBV infection *in vivo*. Flow cytometry was performed to analyze the proportions of F4/80+CD11c+CD206- (M1 macrophages) and F4/80+CD11c-CD206+ (M2 macrophages) macrophages. The results are presented as mean ± standard deviation (*p < 0.05, **p < 0.01, ***p < 0.001, and ****p < 0.0001).

To explore the effect of AA against IBV infection, lung tissue was analyzed using immunofluorescence. We found that more macrophages (green fluorescence, F4/80 protein) were present in the lung tissue of the AA treatment group and this was associated with significant inhibition of IBV replication (red fluorescence, nucleoprotein of IBV) (Figure 5A).

To further analyze the ability of AA to activate macrophages, we performed *in vitro* assays. The isolated bone marrow-derived macrophages (BMMs) were plated on 6-well plates in three groups: AA group, LPS group, and mock control group. Each experimental group was treated with the respective solution and incubated for 48 h. Cells were then collected and stained with F4/80, MHCII, CD40, CD80, and CD86 flow antibodies for flow analysis. Flow cytometry results are shown in Figure 5B. BMMs in the AA-induced group showed greater expression of MHCII compared to the control group (*p* < 0.001). BMMs in the AA-induced group showed increased expression of CD40 (*p* < 0.0001), CD80 (*p* < 0.01), and CD86 (*p* < 0.0001) compared to the mock control group (Figure 5B), indicating that AA-activated macrophages. The above results indicate that Atractyloside A can effectively activate macrophages.

### Atractyloside A can alter macrophage polarization

To analyze the effect of AA on macrophages *in vivo*, IBV-infected mice were euthanized after 7 days and mononuclear cells in the lungs were isolated and analyzed by flow cytometry. We found that the AA treatment group had a reduced proportion of M1 macrophages (F4/80+CD11c+CD206-) compared to the IBV group (*p* < 0.001). The proportion of M2 macrophages (F4/80+CD11c-CD206+) was higher in AA compared to the IBV (*p* < 0.01) and mock groups (*p* < 0.001). We also analyzed the effect of AA on macrophage polarization *in vitro* (Figure 5C), and the results were similar to those *in vivo* (Figure 5D). The above results suggest that AA can regulate macrophage polarization, thereby affecting IBV infection.

## Discussion

Influenza viruses pose a serious threat to human health, infecting hundreds of millions of people worldwide each year with marked impacts on global morbidity and mortality. According to the World Health Organization, the annual seasonal influenza can infect 5–10% of adults and 20–30% of children worldwide, among which 3–5 million are considered to be severe cases (Keech and Beardsworth, 2008; Reed et al., 2015). The related respiratory deaths seriously affect the health of the population and cause huge damage to human health and socioeconomic development every year (Keech and Beardsworth, 2008; Reed et al., 2015). Although vaccination against the influenza virus is one of the most effective preventive strategies against seasonal influenza infections, these vaccines may not adequately protect against new strains due to the high variability and recombination of influenza viruses (Webster et al., 1992; Yoon et al., 2014; Wille and Holmes, 2020). Antiviral drugs play a critical role in stopping the progression and spread of influenza, especially in children, the elderly, and immunocompromised individuals (Han et al., 2018). However, the development of effective vaccines and drugs is challenged by the emergence of new virus subtypes due to constant mutation and genome segment rearrangements (Lyons and Lauring, 2018; Jiang et al., 2020).

At present, the main types of drugs used in the clinical treatment of influenza are NA inhibitors and M2 channel ion blockers (Shen et al., 2015; Yen, 2016; Yin et al., 2021). Among these, oseltamivir, peramivir, and zanamivir are the most commonly utilized NA inhibitors, while the M2 channel ion blockers include drugs such as amantadine and rimantadine (Yin et al., 2021). Recent studies on amantadine treatment effects have shown a significant increase in drug resistance between 2005 and 2007 (Mozhgani et al., 2018); as such, its efficacy against influenza B is inadequate. At present, natural anti-influenza virus drugs remain in the exploratory stages of development. In clinical use, traditional Chinese medicines have shown antiviral activity against the influenza virus without signs of drug resistance (Yin et al., 2021). Studies have provided support for effective antiviral activity in honeysuckle, Radix isatidis, T Terminalia chebula, puerarin, Yin qiao power, and other herbals (Yin et al., 2021).

In this study, the anti-IBV *in vitro* effects of AA were first tested on MDCK and A549 cell lines. Following infection with IBV at 0.1 MOI, we found that the IBV nucleoprotein expression decreased significantly with increased AA concentration (Figures 2A, 3A), indicating that AA can effectively resist IBV infection. To further test whether AA helps to resist IBV infection, we detected HA and the virus copy number. Our results paralleled those from our immunofluorescence and WB analyses; the infectivity of IBV was decreased with an increase in the concentration of AA (Figures 2B, C). Taken together, the above results indicate that AA can help resist IBV infection *in vitro*.

IRF3 is normally present in the cytoplasm in an inactive state. However, in response to viral infection, it is phosphorylated to translocate to the nucleus and activate the transcription of IFNα and IFNβ to mount antiviral infection defenses (Banete et al., 2021). To further analyze the antiviral effects of AA, we analyzed type I interferon-related pathway proteins. We found that after IBV infection of cells, the addition of AA increased the levels of *p*-IRF3 protein (Figure 3B). At the same time, we also found that AA could also increase the levels of *p*-IRF3 in the absence of IBV infection (Supplementary Figure S1). Thus, we used immunofluorescence to test whether *p*-IRF3 could translocate into the nucleus to activate the type I interferon pathway. We found greater *p*-IRF3 in the nucleus after AA was added (Figure 3C). IFN-β-Luc in cells *in vitro* (Figure 3D) and serum INF-β *in vivo* (Figure 3E) both indicated that AA can effectively induce the production of type I interferon. Currently, a large number of studies have shown that type 1 interferon is an effective viral inhibitor. Our work suggests that AA can increase the phosphorylation of IRF3 to induce the expression of IFN-β to resist IBV infection. To further demonstrate this, it is necessary to block the IFN receptor or IFN production to observe whether the AA effects are eliminated. Unfortunately, we did not conduct this experiment in the present study. Therefore, we can only speculate that AA activates the type I interferon pathway to inhibit the replication of IBV. We will further analyze the relationship between AA and type I interferon antiviral in subsequent experiments.

Our *in vivo* study of the anti-IBV effects of oral AA administration showed that drug administration was associated with improvements in body weight and food intake and other signs of IBV infection. The IBV-infected group had a protection rate of 62.5% after infection, and survival was significantly improved (to 100%) with AA treatment (Figure 4D). Our post-viral lung pathology analyses showed that AA can effectively attenuate the lung damage caused by the influenza virus and reduce the potential damage of viral pneumonia (Figures 4E, F). At the same time, we also performed an immunohistochemical analysis of IBV nucleoprotein and found that AA can effectively inhibit IBV in the lungs. The above results showed that AA can resist IBV infection.

Macrophages are important immunoregulatory cells that play a critical role in the development of inflammation and responses to viral infection including the development of pneumonia. Therefore, we tested AA's effects on macrophage activation and polarization. We isolated primary bone marrow-derived BMMs and performed flow cytometry analysis to assess macrophage responses to AA administration. We found that AA effectively induced the expression of MHCII and the costimulatory molecules CD40, CD80, and CD86 on BMMs (Figure 5B). Activation of macrophages can facilitate virus recognition and immunomodulation that then facilitates T-cell responses to the pathogen (Banete et al., 2021). These results indicate that AA can effectively activate macrophages to phagocytose and deal with pathogens.

Macrophage polarization changes occur in response to environmental stimuli; M1-type polarization of activated macrophages is associated with Th1 cytokines, and M2-type macrophage polarization is associated with Th2 cytokines (Yunna et al., 2020; Banete et al., 2021). Therefore, we analyzed the effect of AA on macrophage polarization in lung tissue. We found that after infection with IBV, the proportion of M1-type macrophages in the AA-treated group was significantly reduced, while the proportion of M2-type macrophages was increased (Figure 5C). Previous studies have shown that M1-type macrophages can facilitate the ability of influenza viruses to use the inflammatory response to support their replication, while M2-type macrophages can have anti-inflammatory and tissue repair-promoting effects (Banete et al., 2021). Therefore, we infer that AA can reduce the M1-type polarization of macrophages, reduce the inflammatory response, and inhibit the replication of the influenza virus in the lung. By increasing M2 polarization, AA may promote anti-inflammatory and lung tissue repair mechanisms.

In conclusion, in this study, the inhibitory effects of AA on IBV were explored by utilizing *in vivo* and *in vitro* models. We found that AA has anti-IBV effects. In addition, we identified novel AA effects on macrophage function in the context of IBV infection. This study provides a new theoretical basis for the development of anti-IBV drugs.

## Data availability statement

The original contributions presented in the study are included in the article/Supplementary material, further inquiries can be directed to the corresponding authors.

## Ethics statement

The animal study was reviewed and approved by Institutional Animal Care and Use Committee (IACUC) of the Changchun University of Chinese Medicine.

## Author contributions

DL, WL, HL, and JH were responsible for the experiment design and drafting of the manuscript. JH, XZ, ZG, PW, JZ, YX, and YuL performed experiments. JH, JF, YiL, and YZ analyzed data. All authors contributed to the article and approved the submitted version.

## Supplementary material

Supplementary Figure S1 Effect of AA on the expression of IRF3 and *p*-IRF3. **(A)** Western blot analysis of IRF3 and *p*-IRF3 protein expression changes after Atractyloside A treatment of A549.

## References

[B1] Al-Karmalawy A. A. Soltane R. Abo Elmaaty A. Tantawy M. A. Antar S. A. Yahya G.. (2021). Coronavirus disease (COVID-19) control between drug repurposing and vaccination: a comprehensive overview. Vaccines (Basel) 9. 10.3390/vaccines9111317 34835248 PMC8622998

[B2] Banete A. Barilo J. Whittaker R. Basta S. (2021). The activated macrophage - a tough fortress for virus invasion: how viruses strike back. Front. Microbiol. 12, 803427. 10.3389/fmicb.2021.803427 35087503 PMC8787342

[B3] Belongia E. A. Simpson M. D. King J. P. Sundaram M. E. Kelley N. S. Osterholm M. T.. (2016). Variable influenza vaccine effectiveness by subtype: a systematic review and meta-analysis of test-negative design studies. Lancet Infect. Dis. 16, 942–951. 10.1016/S1473-3099(16)00129-8 27061888

[B4] Feng L. Z. Peng Z. B. Wang D. Y. Yang P. Yang J. Zhang Y. Y.. (2018). Technical guidelines for seasonal influenza vaccination in China, 2018-2019. Zhonghua Liu Xing Bing Xue Za Zhi 39, 1413–1425. 30462947 10.3760/cma.j.issn.0254-6450.2018.11.001

[B5] Ferdinands J. M. Fry A. M. Reynolds S. Petrie J. Flannery B. Jackson M. L.. (2017). Intraseason waning of influenza vaccine protection: evidence from the US Influenza Vaccine Effectiveness Network, 2011-12 through 2014-15. Clin. Infect. Dis. 64, 544–550. 10.1093/cid/ciw816 28039340

[B6] Han J. Perez J. Schafer A. Cheng H. Peet N. Rong L.. (2018). Influenza virus: small molecule therapeutics and mechanisms of antiviral resistance. Curr. Med. Chem. 25, 5115–5127. 10.2174/0929867324666170920165926 28933281 PMC8735713

[B7] Han J. C. Li Q. X. Fang J. B. Zhang J. Y. Li Y. Q. Li S. Z.. (2021). GII.P16-GII.2 recombinant norovirus VLPs polarize macrophages into the M1 phenotype for Th1 immune responses. Front. Immunol. 12, 781718. 10.3389/fimmu.2021.781718 34868056 PMC8637406

[B8] Huang W. J. Cheng Y. H. Tan M. J. Liu J. Li X. Y. Zeng X. X.. (2022). Epidemiological and virological surveillance of influenza viruses in China during 2020-2021. Infect. Dis. Poverty 11, 74. 10.1186/s40249-022-01002-x 35768826 PMC9244124

[B9] Jackson M. L. Chung J. R. Jackson L. A. Phillips C. H. Benoit J. Monto A. S.. (2017). Influenza vaccine effectiveness in the United States during the 2015-2016 season. N. Engl. J. Med. 377, 534–543. 10.1056/NEJMoa1700153 28792867 PMC5727917

[B10] Jiang D. Wang Q. Bai Z. Qi H. Ma J. Liu W.. (2020). Could environment affect the mutation of H1N1 influenza virus? Int. J. Environ. Res. Public Health 17. 10.3390/ijerph17093092 32365515 PMC7246512

[B11] Keech M. Beardsworth P. (2008). The impact of influenza on working days lost: a review of the literature. Pharmacoeconomics 26, 911–924. 10.2165/00019053-200826110-00004 18850761

[B12] Lyons D. M. Lauring A. S. (2018). Mutation and epistasis in influenza virus evolution. Viruses 10. 10.3390/v10080407 30081492 PMC6115771

[B13] Mozhgani S. H. Zarei Ghobadi M. Moeini S. Pakzad R. Kananizadeh P. Behzadian F.. (2018). Prevalence of human influenza virus in Iran: evidence from a systematic review and meta-analysis. Microb. Pathog. 115, 168–174. 10.1016/j.micpath.2017.12.064 29284132

[B14] Paules C. Subbarao K. (2017). Influenza. Lancet 390, 697–708. 10.1016/S0140-6736(17)30129-0 28302313

[B15] Paules C. I. Sullivan S. G. Subbarao K. Fauci A. S. (2018). Chasing seasonal influenza - the need for a universal influenza vaccine. N. Engl. J. Med. 378, 7–9. 10.1056/NEJMp1714916 29185857

[B16] Petrova V. N. Russell C. A. (2018). The evolution of seasonal influenza viruses. Nat. Rev. Microbiol. 16, 60. 10.1038/nrmicro.2017.118 29109554

[B17] Reed C. Chaves S. S. Daily Kirley P. Emerson R. Aragon D. Hancock E. B.. (2015). Estimating influenza disease burden from population-based surveillance data in the United States. PLoS ONE 10, e0118369. 10.1371/journal.pone.0118369 25738736 PMC4349859

[B18] Shaw M. W. Xu X. Li Y. Normand S. Ueki R. T. Kunimoto G. Y.. (2002). Reappearance and global spread of variants of influenza B/Victoria/2/87 lineage viruses in the 2000-2001 and 2001-2002 seasons. Virology 303, 1–8. 10.1006/viro.2002.1719 12482653

[B19] Shen Z. Lou K. Wang W. (2015). New small-molecule drug design strategies for fighting resistant influenza A. Acta. Pharm. Sin. B. 5, 419–430. 10.1016/j.apsb.2015.07.006 26579472 PMC4629447

[B20] Tu J. Xie Y. Xu K. Qu L. Lin X. Ke C.. (2020). Treatment of spleen-deficiency syndrome with Atractyloside A from bran-processed Atractylodes lancea by protection of the intestinal mucosal barrier. Front. Pharmacol. 11, 583160. 10.3389/fphar.2020.583160 33658928 PMC7919195

[B21] Uyeki T. M. (2017). Influenza. Ann. Intern. Med. 167, ITC33–ITC48. 10.7326/AITC201709050 28869984

[B22] Webster R. G. Bean W. J. Gorman O. T. Chambers T. M. Kawaoka Y. (1992). Evolution and ecology of influenza A viruses. Microbiol. Rev. 56, 152–179. 10.1128/mr.56.1.152-179.1992 1579108 PMC372859

[B23] Wille M. Holmes E. C. (2020). The ecology and evolution of influenza viruses. Cold Spring. Harb. Perspect. Med. 10. 10.1101/cshperspect.a038489 31871237 PMC7328453

[B24] Yamayoshi S. Kawaoka Y. (2019). Current and future influenza vaccines. Nat. Med. 25, 212–220. 10.1038/s41591-018-0340-z 30692696 PMC12973209

[B25] Yen H. L. (2016). Current and novel antiviral strategies for influenza infection. Curr. Opin. Virol. 18, 126–134. 10.1016/j.coviro.2016.05.004 27344481

[B26] Yin H. Jiang N. Shi W. Chi X. Liu S. Chen J. L.. (2021). Development and effects of influenza antiviral drugs. Molecules 26. 10.3390/molecules26040810 33557246 PMC7913928

[B27] Yoon S. W. Webby R. J. Webster R. G. (2014). Evolution and ecology of influenza A viruses. Curr. Top Microbiol. Immunol. 385, 359–375. 10.1007/82_2014_396 24990620

[B28] Yunna C. Mengru H. Lei W. Weidong C. (2020). Macrophage M1/M2 polarization. Eur. J. Pharmacol. 877, 173090. 10.1016/j.ejphar.2020.173090 32234529

