# Supplementary figures and images for: Antiviral effects of Atractyloside A on the influenza B virus (Victoria strain) infection
Source: Front Microbiol. 2023 Jan 10;13:1067725. doi: 10.3389/fmicb.2022.1067725 (PMC9871751; doi:10.3389/fmicb.2022.1067725)

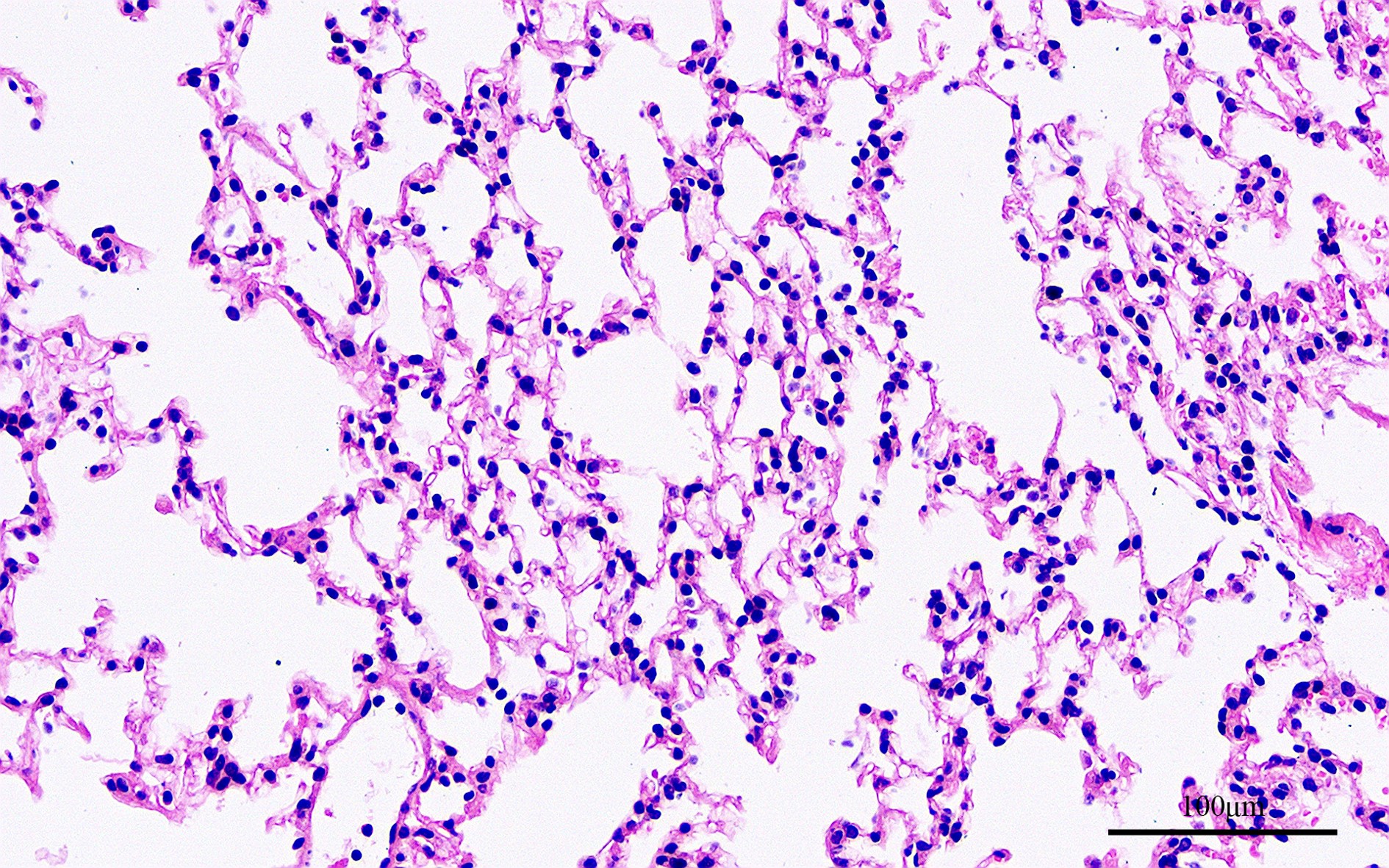

Supplement: Supplementary file 1 [file Data_Sheet_1.ZIP › Original data/HE/FIG.4F/CONTROL.jpg]

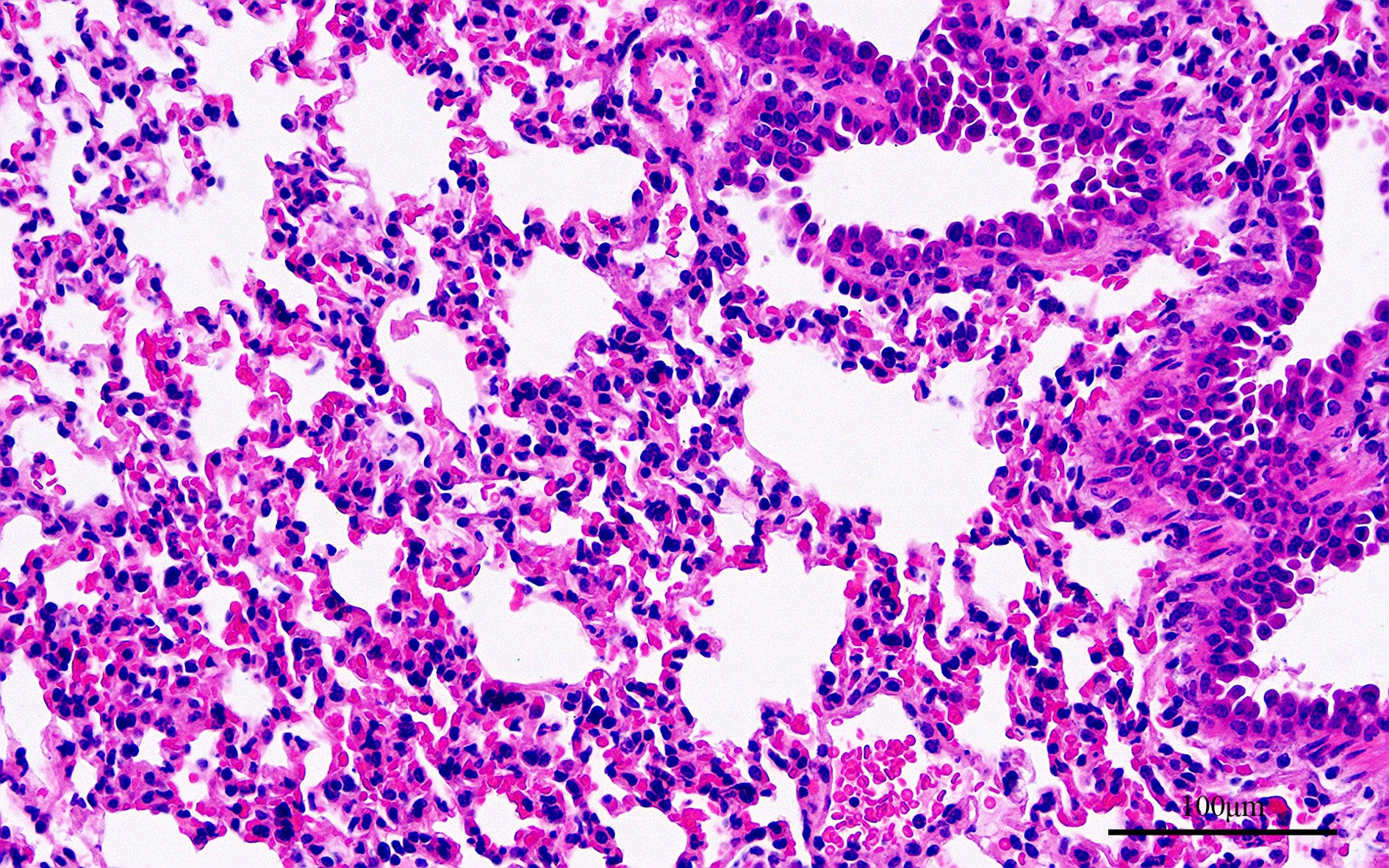

Supplement: Supplementary file 1 [file Data_Sheet_1.ZIP › Original data/HE/FIG.4F/IBV+AA10.jpg]

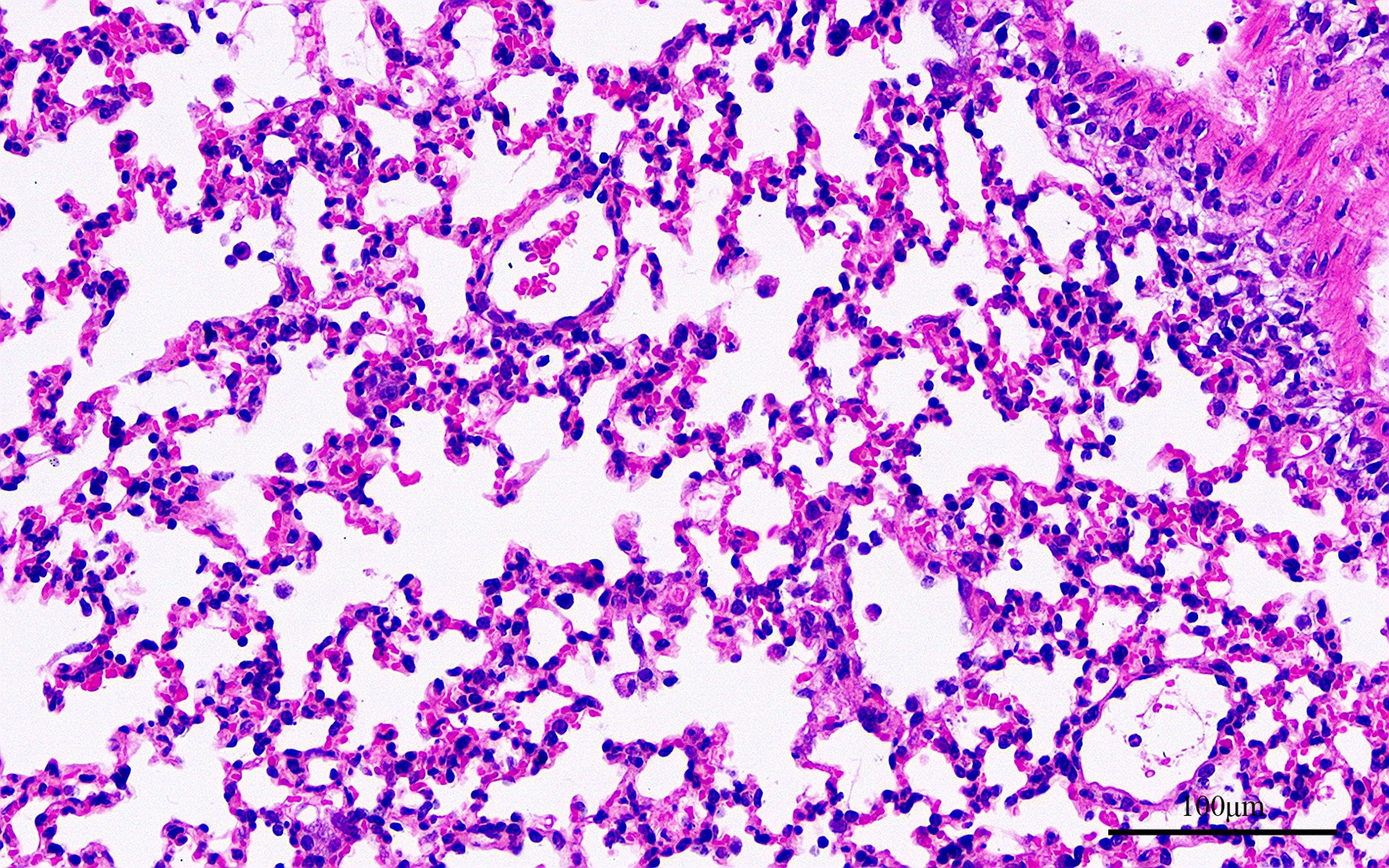

Supplement: Supplementary file 1 [file Data_Sheet_1.ZIP › Original data/HE/FIG.4F/IBV+AA30.jpg]

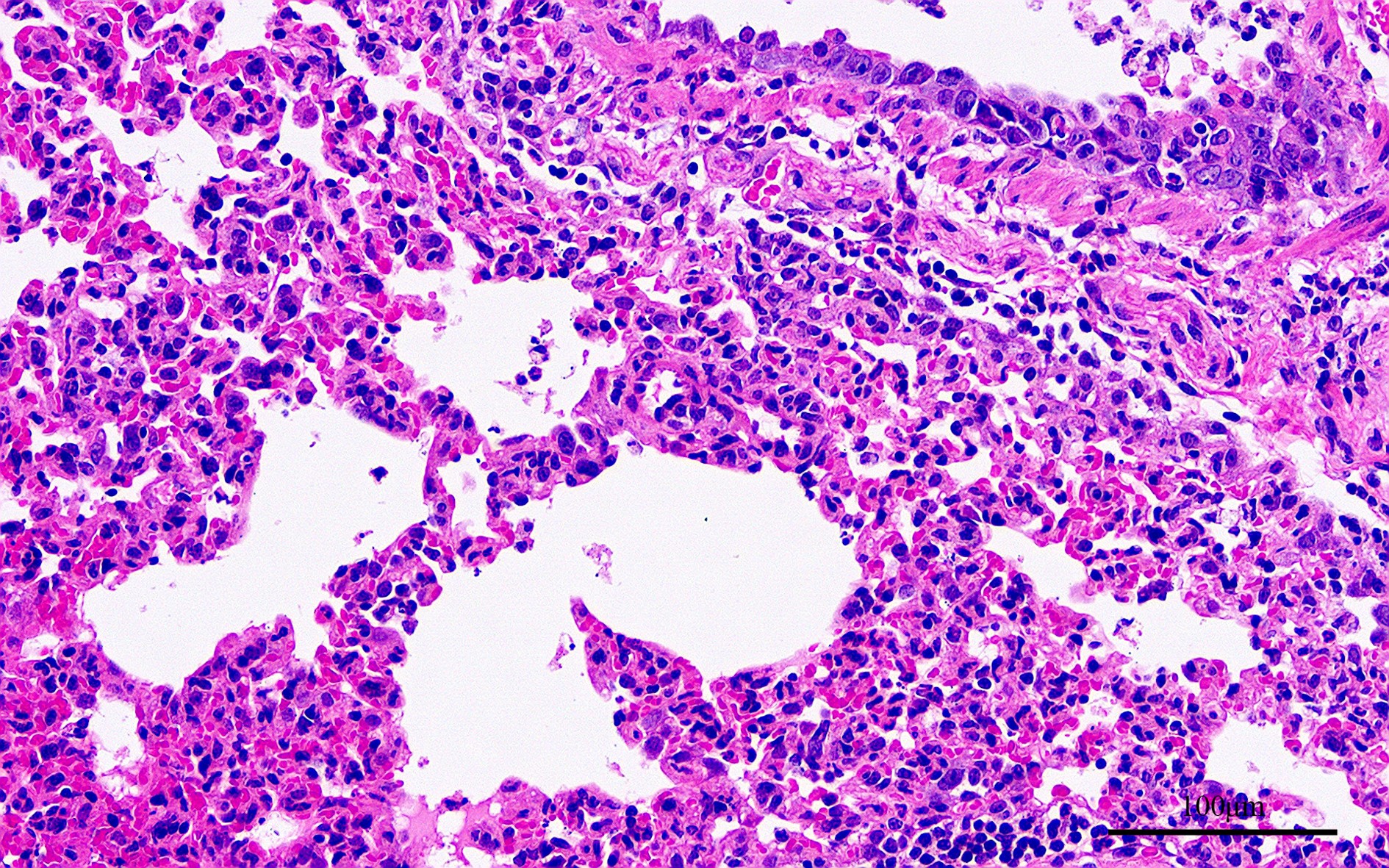

Supplement: Supplementary file 1 [file Data_Sheet_1.ZIP › Original data/HE/FIG.4F/IBV.jpg]

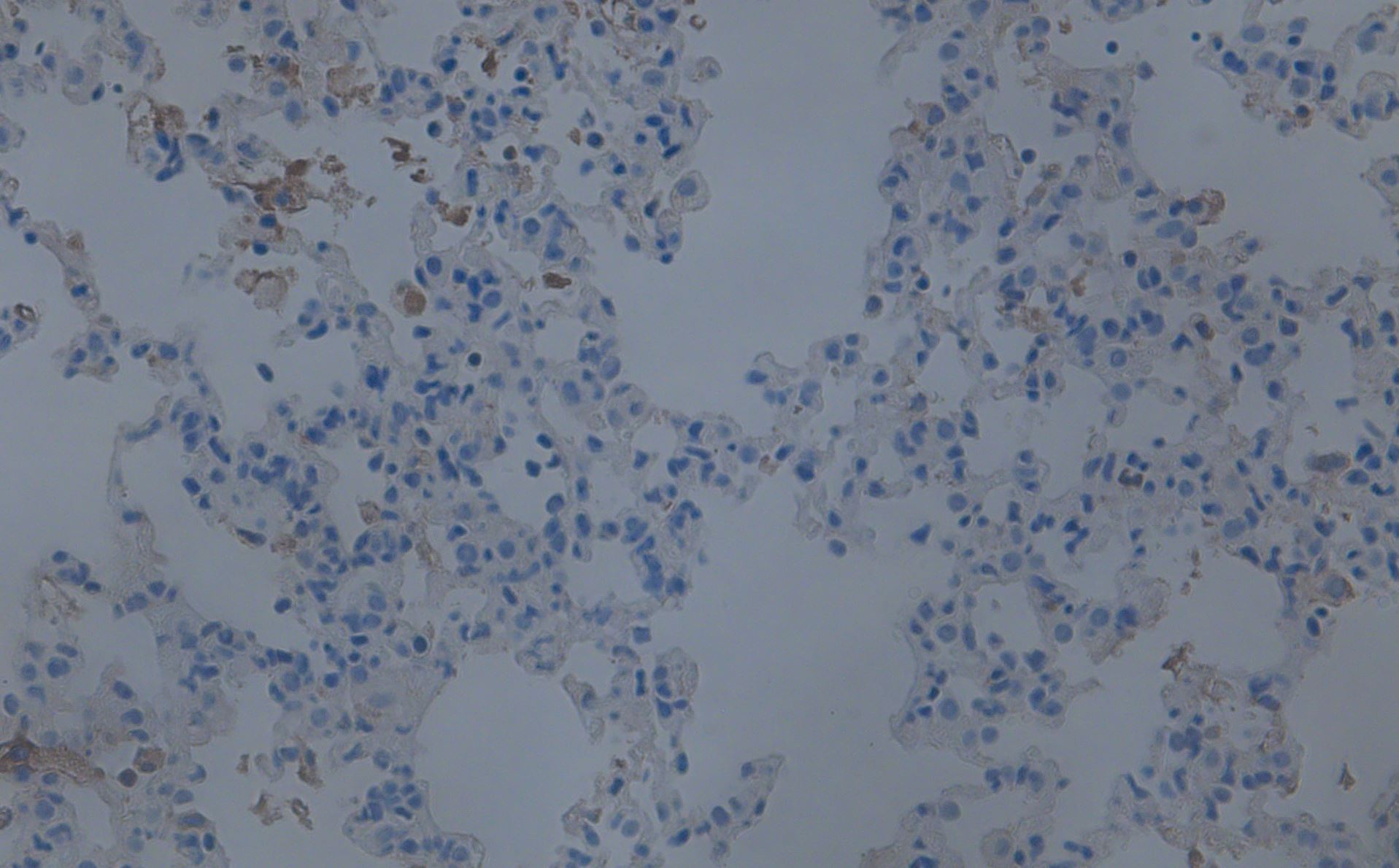

Supplement: Supplementary file 1 [file Data_Sheet_1.ZIP › Original data/Immunohistochemistry/FIG.4G/IBV+AA10.JPG]

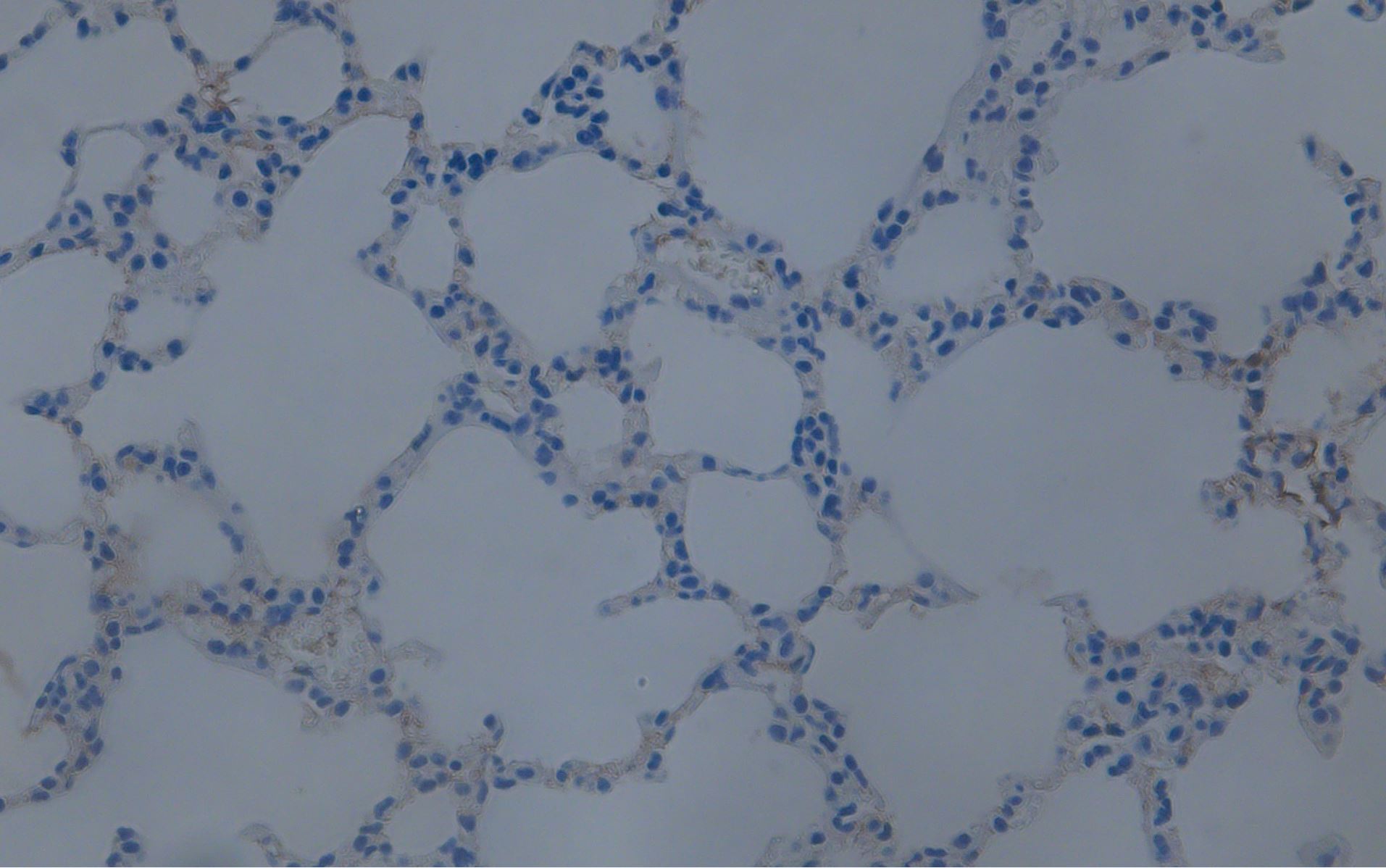

Supplement: Supplementary file 1 [file Data_Sheet_1.ZIP › Original data/Immunohistochemistry/FIG.4G/IBV+AA30.JPG]

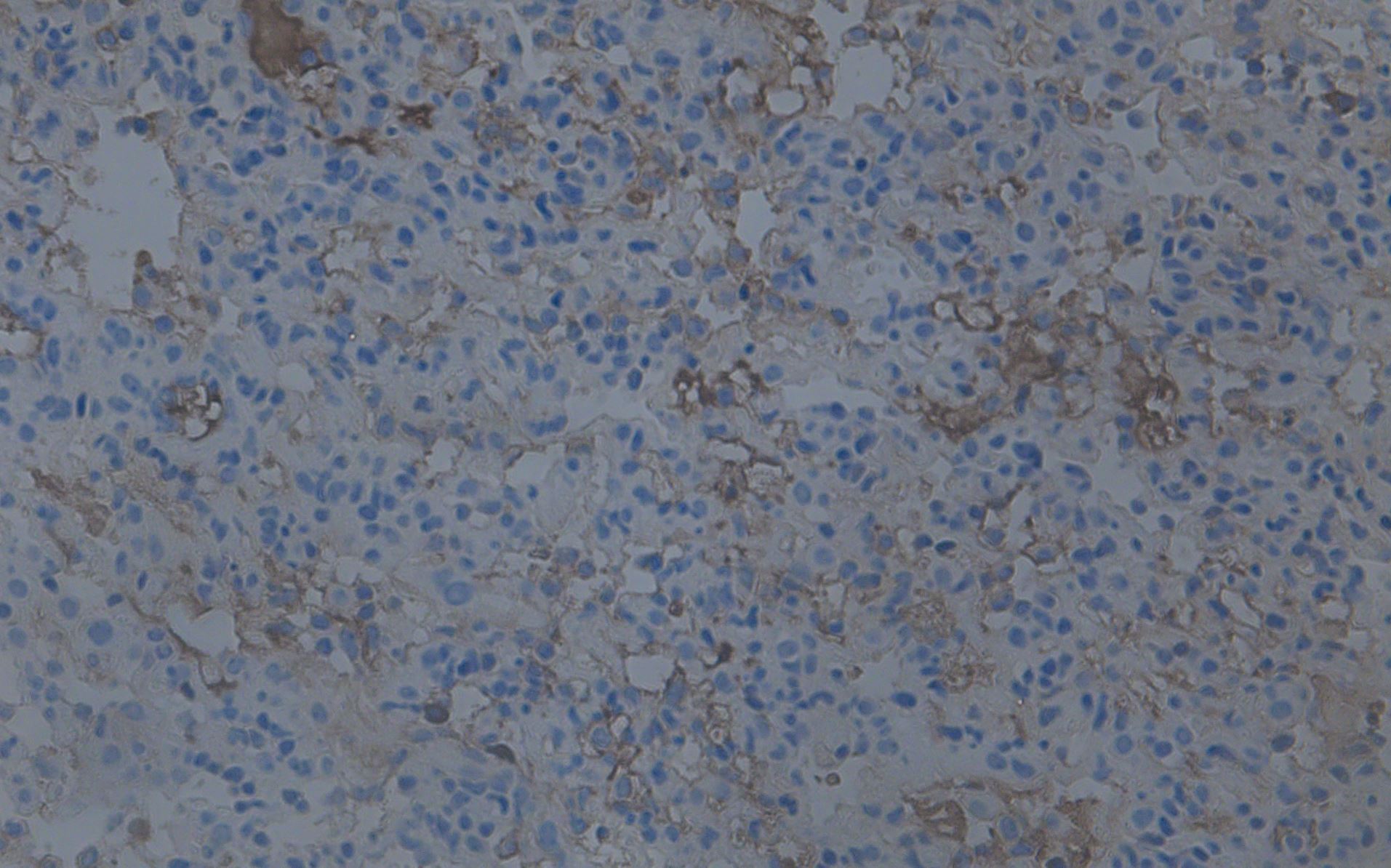

Supplement: Supplementary file 1 [file Data_Sheet_1.ZIP › Original data/Immunohistochemistry/FIG.4G/IBV.JPG]

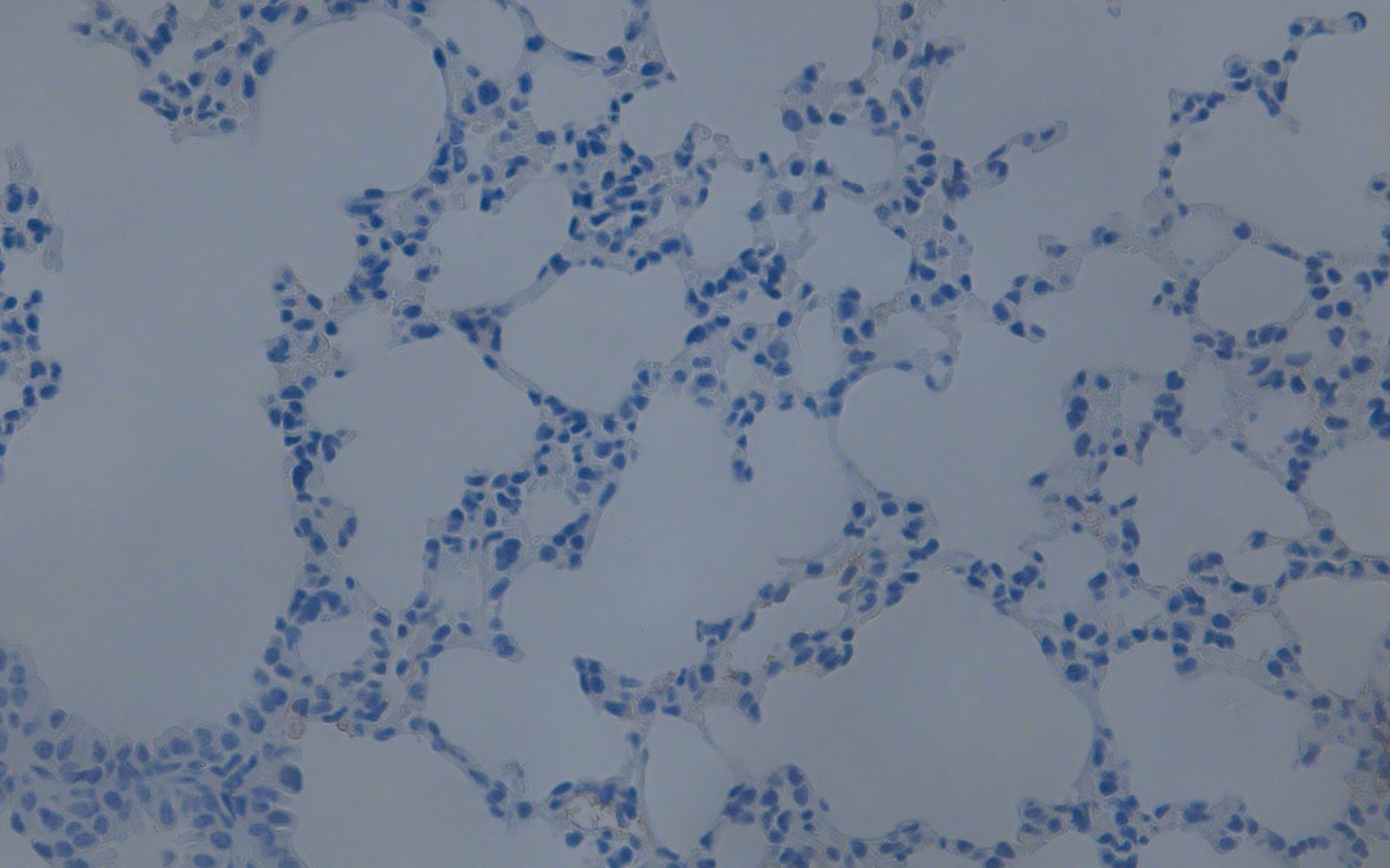

Supplement: Supplementary file 1 [file Data_Sheet_1.ZIP › Original data/Immunohistochemistry/FIG.4G/control.JPG]

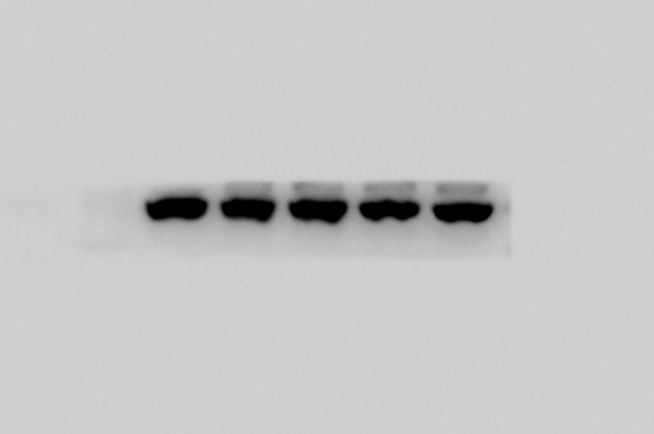

Supplement: Supplementary file 1 [file Data_Sheet_1.ZIP › Original data/westernblot/FIG.3B/GAPDH.JPG]

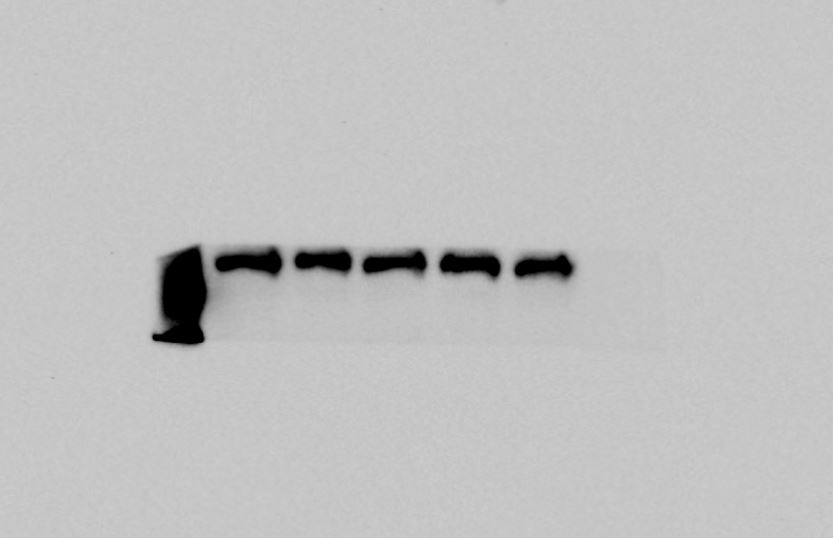

Supplement: Supplementary file 1 [file Data_Sheet_1.ZIP › Original data/westernblot/FIG.3B/IRF3.JPG]

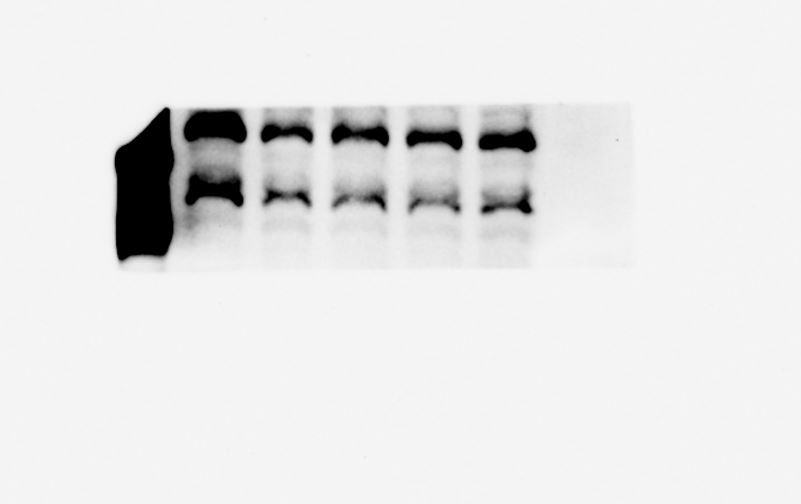

Supplement: Supplementary file 1 [file Data_Sheet_1.ZIP › Original data/westernblot/FIG.3B/MAVS.JPG]

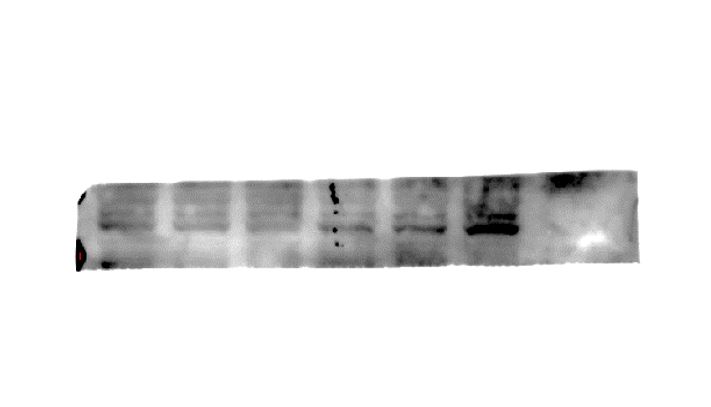

Supplement: Supplementary file 1 [file Data_Sheet_1.ZIP › Original data/westernblot/FIG.3B/P-IRF3.JPG]

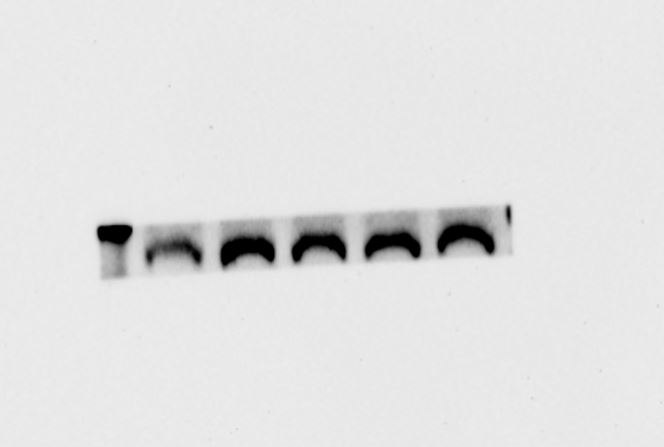

Supplement: Supplementary file 1 [file Data_Sheet_1.ZIP › Original data/westernblot/FIG.3B/P-TBK1.JPG]

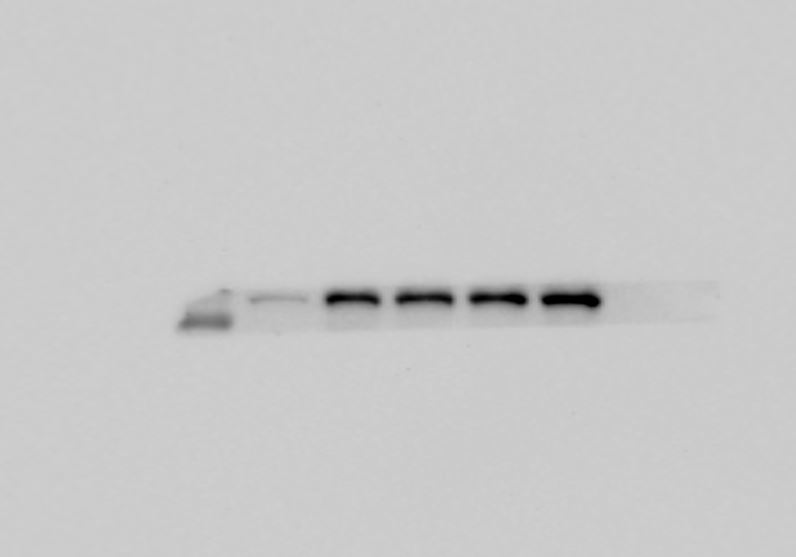

Supplement: Supplementary file 1 [file Data_Sheet_1.ZIP › Original data/westernblot/FIG.3B/RIG-I.JPG]

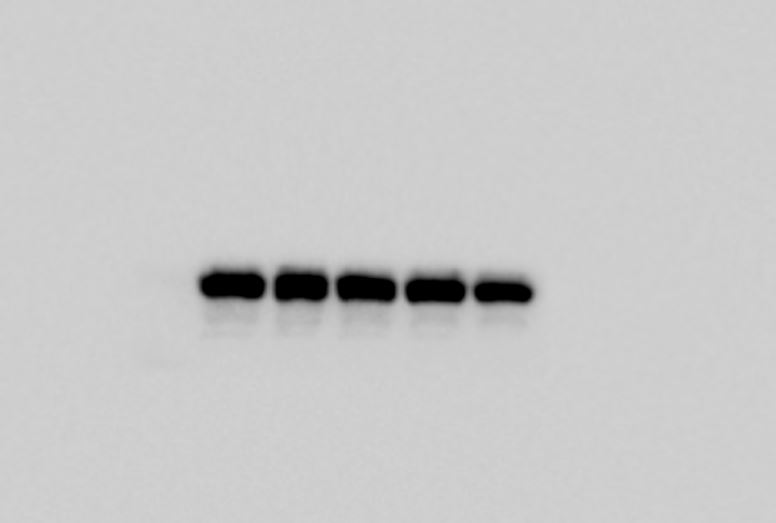

Supplement: Supplementary file 1 [file Data_Sheet_1.ZIP › Original data/westernblot/FIG3.A/GAPDH.JPG]

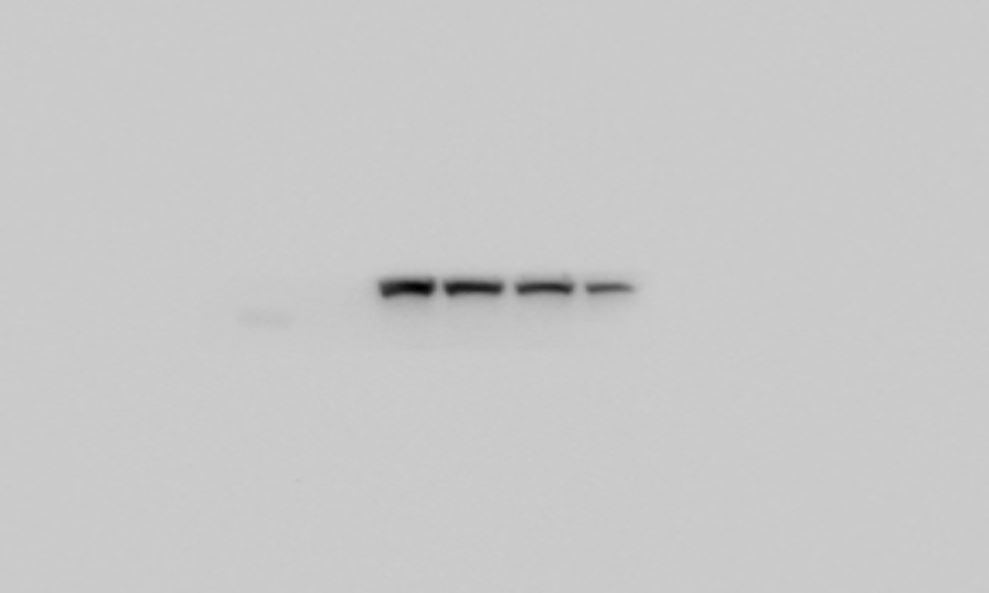

Supplement: Supplementary file 1 [file Data_Sheet_1.ZIP › Original data/westernblot/FIG3.A/IBV-NP.JPG]

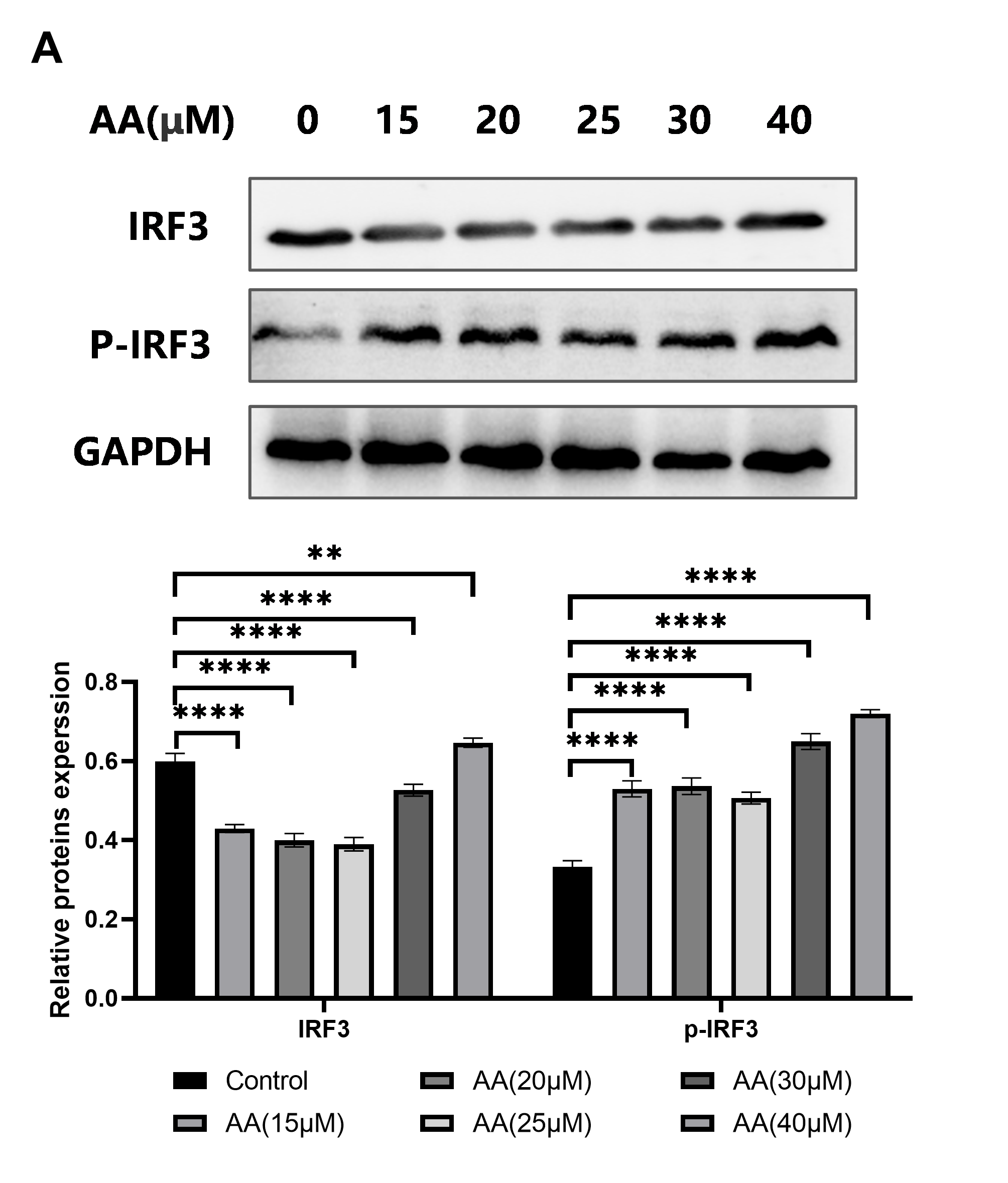

Supplement: Supplementary Figure S1 — Effect of AA on the expression of IRF3 and p-IRF3. (A) Western blot analysis of IRF3 and p-IRF3 protein expression changes after Atractyloside A treatment of A549. [file Image_1.TIF]
